# Pathway-Driven Discovery of Rare Mutational Impact on Cancer

**DOI:** 10.1155/2014/171892

**Published:** 2014-05-04

**Authors:** TaeJin Ahn, Taesung Park

**Affiliations:** ^1^Interdisciplinary Program in Bioinformatics, Seoul National University, San 56-1, Shilim-dong, Kwanak-gu, Seoul 151-742, Republic of Korea; ^2^Samsung Genome Institute, Samsung Medical Center, Irwon-ro 81, Seoul 136-710, Republic of Korea; ^3^Department of Statistics, Seoul National University, San 56-1, Shilim-dong, Kwanak-gu, Seoul 151-742, Republic of Korea

## Abstract

Identifying driver mutation is important in understanding disease mechanism and future application of custom tailored therapeutic decision. Functional analysis of mutational impact usually focuses on the gene expression level of the mutated gene itself. However, complex regulatory network may cause differential gene expression among functional neighbors of the mutated gene. We suggest a new approach for discovering rare mutations that have real impact in the context of pathway; the philosophy of our method is iteratively combining rare mutations until no more mutations can be added under the condition that the combined mutational event can statistically discriminate pathway level mRNA expression between groups with and without mutational events. Breast cancer patients with somatic mutation and mRNA expression were analyzed by our approach. Our approach is shown to sensitively capture mutations that change pathway level mRNA expression, concurrently discovering important mutations previously reported in breast cancer such as TP53, PIK3CA, and RB1. In addition, out of 15,819 genes considered in breast cancer, our approach identified mutational events of 32 genes showing pathway level mRNA expression differences.

## 1. Introduction

Cancer starts from normal cells, acquires mutations, and evolves to be malignant cancer cells metastatic and/or resistant to therapy. Recent development of next generation sequencing technology has revealed many somatic mutations from individuals; most of them are rare and lack functional information.

Mutation information provides crucial hints in cancer medicine. For example, KRAS mutation is a test recommended for targeted drug response of colon cancer therapy [1]. EGFR activation mutations and resistant mutations are another recommended gene mutation check for targeted therapy of nonsmall cell lung cancers [2]. However, only a few mutations are known to be clinically actionable, and most of less frequent mutations remain obscure.

Mutation information in conjunction with mRNA pathway level alteration can help to customize a patient's medicine. In the study of Jones et al. [3], a patient had metastasized tumor in lung after surgery of his primary site. Biopsy from his lung tumor had sequenced for mutation and transcription profiling. Pathway analysis based on differentially expressed genes, in addition to integrated CNV and mutation information, helped doctor's decision to change his drug, which stabilized his disease for three months.

To measure the pathway level impact of rare mutations from an individual sample, a method should measure individual's pathway level aberrance. Several researches have tackled this issue. PARADIGM is a tool that infers a pathway status by using a known functional structure [4]. PARADIGM models functional structure of pathway as a set of interconnected variables where variables are omics objects like DNA, mRNA, and protein. Interaction between variables describes functional status of pathway. PARADIGM might perform better to quantify pathway level with multiple omics; it utilizes known functional relationship between genes' and intergenes' DNA to protein. Hence, the performance in a single layer omics data, handling only mRNA microarray, may not be promising.

Drier et al. proposed a personal pathway deregulation score (PDS) representing the distance of a single cancer sample from median of normal samples on the principal curve [5]. To calculate PDS, Drier et al. reduce the dimensions by PCA and find the best principal curve, utilizing entire cohort samples which contain both normal and different stages of cancers. Drier et al.'s method performs well in the mRNA only data sets, brain, and colon cancer, than PARADIGM does.

In our unpublished previous study [6], we proposed pathway statistics to reflect individual's pathway aberrance (individualized pathway aberrance score, iPAS) by modifying existing pathway statistics that can further be categorized as overrepresenting analysis approach (ORA) or functional class scoring (FCS) [7].

ORA typically apply an arbitrary threshold (e.g., fold-change > 2 or *P* value < 0.05) on gene expression to assess if the number of genes beyond threshold is significantly over- or underrepresented in the given pathway. There are two drawbacks known for ORA. First, it only uses the most significant genes and discards the others, thus resulting in information loss of marginally significant genes. Second, it only considers the number of genes and does not consider extent of expression changes, thus causing another information loss of importance of genes (e.g., genes fold-change = 2.01 and fold-change = 4 are considered equally). Unlike ORA, FCS methods do not discard genes with arbitral threshold but use all available genes showing improvements over ORA. Method based on pathway topology has been known to compensate common limitations of ORA and FCS reporting false positive gene sets, due to the set of overlapping genes.

The key idea of our previous study [6] is comparing a single tumor sample to many reference normal samples to provide gene level statistics for FCS and ORA analysis. The approach demonstrated that it not only captures previously known biological knowledge but also reveals pathway based sample clusters that show clinically important associations such as cancer differentiation and patient survival. In this paper, we adopt the best-performing pathway statistic of the previous study for summarizing an individual patient's pathway expression level.

We suggest an integrated analysis of mutation and gene expression to discover a combination of rare mutations that causes pathway level gene expression changes. We applied our method to the cancer genome atlas (TCGA) [8] breast cancer data providing both somatic mutation and mRNA expression data from the same patients (*n* = 513). Assuming that combination of functionally related rare mutations can influence the pathway of mRNA expression, we consider multiple rare mutations to be counted as a single mutational event. At the first step of Algorithm 1, we assess if pathway level mRNA expression is significantly different between groups with and without mutational events. If it is different, we add another mutation site to be counted as a single mutational event and then assess if the new event can still differentiate pathway level mRNA expression. We iterate this procedure until no more rare mutation can be added into mutational event under the certain significance threshold for pathway level gene expression difference. The identified mutations can be biologically interpreted as a set of mutations that influence the pathway level gene expression, and thus the mutations can be considered functional in cancer, which can be further prioritized as cancer drivers.

Our empirical study demonstrated that highly frequent single gene mutations on TP53 or PIK3CA are strong enough to show pathway level mRNA expression difference between mutated and nonmutated groups, respectively. We also found combinations of less frequent mutations having impact on pathway level gene expression. Most of the mutations in our discovery did not show gene level expression difference between mutated samples and wild type samples, suggesting that pathway level gene expression change is beneficial to discover mutations with functional impact. It is important to note that our discovery is concordantly capturing the TP53, PIK3CA, and RB mutations that have been importantly discussed in breast cancer pathways [8]. This suggests that our approach is useful to discover potential cancer driver mutations.

## 2. Materials and Methods

### 2.1. Mutation and Gene Expression Data

Somatic mutation (WUSM mutation calling) and normalized gene expression (UNC Agilent G4502A_07, level 3) data of breast cancer are downloaded from TCGA website (The Cancer Genome Atlas Network, 2012). The level 3 TCGA mRNA data provide gene level summary of mRNA expression, which is standardized by mean and standard deviation of entire dataset. Samples having both mutation and gene expression data (*n* = 513) are used for analysis. Missing gene expressions are replaced by the mean of gene expression of normal tissues. This replacement means that the replaced missing value will give no contribution in a positive way or negative way in pathway level gene summary; literally its value becomes zero after it is standardized by the mean and standard deviation of normal samples.

### 2.2. Pathway Data

Pathway gene sets are downloaded from molecular signature database. Total 583 gene sets from BioCarta, NCI cancer pathway, and KEGG pathway are used for our analysis. We also manually defined additional gene sets to assess rare mutations' impact on pathways that include more than one drug target; we defined 16 receptor tyrosine kinase (RTK) genes which have more than one approved targeted drugs. Associated drug information is retrieved from Ingenuity (www.ingenuity.com). For each of RTK genes, we expanded the gene set by adding its first neighbors by adopting protein-protein interaction data in HipathDB [8]. Through this annotation process, we obtained gene sets representing 16 RTK pathways. Summary of RTK pathways used in our study is provided in Table 1.

### 2.3. Individualized Pathway Statistic

Standardizing gene expression by mean and standard deviation from data set is often used for microarray analysis [9–13]. A vector *z* = (*z*_1_, *z*_2_, …, *z*_*n*_) denotes expression status of a pathway where *z*_*i*_ symbolizes the standardized expression value of the *i*th gene, where the number of genes that belong to the pathway is *n*. In our settings, standardization is performed by mean and standard deviation (s.d.) of data set from normal tissue of cancer patients. Thus, avg(*z*)/*n* is indicating how much the given individual cancer sample's overall gene expression of the pathway deviates from the center of normal tissue data set.

### 2.4. Iterative Assessment of Mutation Impact on Pathway

In our analysis, multiple rare mutations on the gene(s) are defined as a single mutational event; in other words, we consider multiple mutations on the single gene as a unit of mutational event. Initially, our algorithm calculates a single gene's mutational impact in the pathway level mRNA expression change. In the loop, it iteratively adds genes to mutational event while the mutational event induces significant difference between the two groups, with mutation and without mutation. As the iteration continues, the overall number of significance tests is recorded and used to calculate the* q* value. The algorithm generates a set of genes as an output. The output can be interpreted as a set of rare mutations from a (several) gene(s) that have significant pathway level gene expression difference. Pseudocode description of our procedure is as follows. Algorithm after the “for” loop (5~20) is schematically described in Supplementary Figure 1 ( see Supplementary Material available online at http://dx.doi.org/10.1155/2014/171892).

## 3. Results

**Figure 1:**
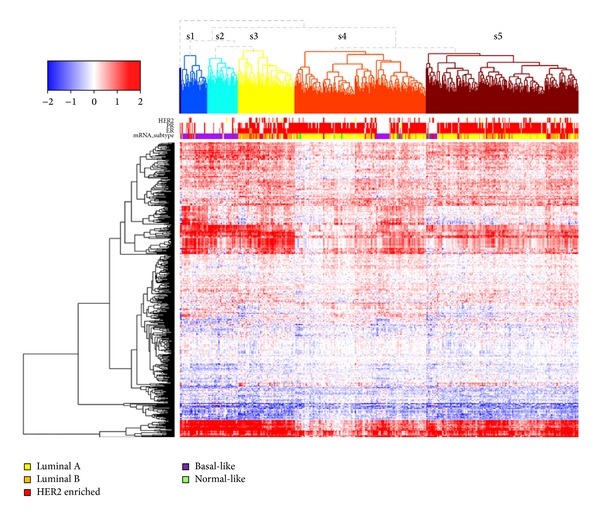
Clustering 513 TCGA breast cancer cases by individualized pathway score. Each row represents a pathway, each column represents a sample.

**Figure 2:**
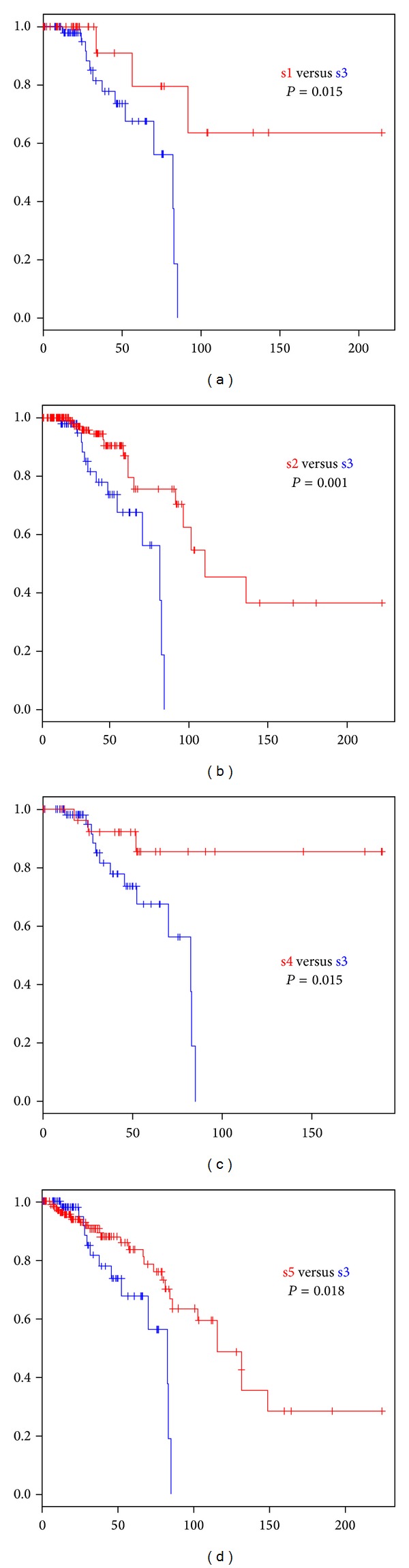
Survival difference by sample cluster subtype of pathway score based clustering.

Individual pathway score based clustering of 513 breast cancer samples revealed 5 sample clusters. Sample cluster represents mRNA expression based subtype of breast cancer. Luminal A subtype is dominant at sample subgroup s4 and s5. Basal-like subtype is enriched at sample subgroup s1 and s2. Basal-like subtype in subgroup s4 can be distinguished by ER-, PR-, and HER2-. Sample cluster s3 is enriched by luminal B subtype (Figure 1). Sample cluster s3 shows the most unfavorable outcome when it is compared to other subtypes (Figure 2: *P* = 0.015 for s3 versus s1, *P* = 0.015 for s3 versus s2, *P* = 0.001 for s3 versus s4, and *P* = 0.018 for s3 versus s5). This finding is concordant with the previously reported biological knowledge that luminal B subtype has the worst outcome among mRNA expression based subtypes of breast cancer [14]. The representation of mRNA based cancer subtype by clustering the pathway scores indicates that our individualized pathway scoring method indeed captures the clinical characteristic of each cancer samples. The result satisfied us to further utilize our approach to assess rare mutations' impact on pathway level gene expression.

**Figure 3:**
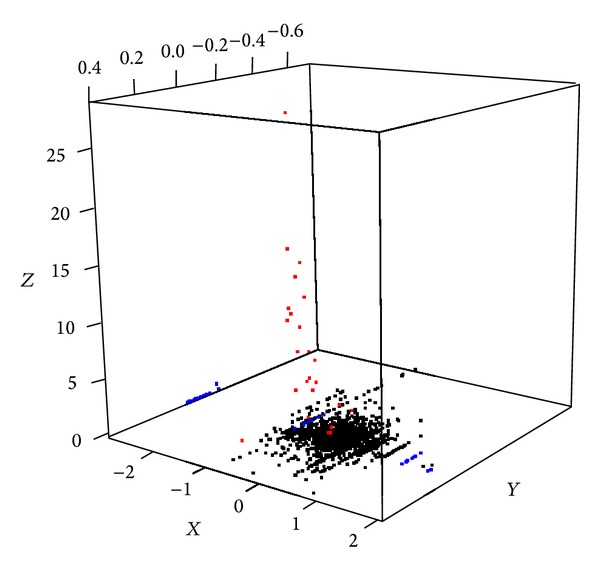
Single gene's mutational influence on mRNA expression at gene level (*X* axis) and pathway level (*Y* axis).* X*: averaged gene expression difference of mutation having group minus nonhaving group).* Y*: averaged pathway level difference of mutation having group minus nonhaving group).* Z*: −log10*p* score, where* p* is from* t*-test of pathway statistics between mutated group versus nonmutated group. Red: mutation event where its influence on pathway level is significant (FDR* q* value < 0.1). Blue dots: mutation event where its influence on gene level is significant (FDR* q* value < 0.1) but not significant at pathway level.

To evaluate whether our method can sensitively capture the impact of mutation event on the pathway level gene expression, we analyzed breast cancer data set from TCGA having paired somatic mutation and expression data (*n* = 513). Mutation event of single gene is described in Figure 3. Mutations that change gene expression level of the mutated genes (FDR* q* value < 0.1) are shown in blue. Mutations that do not change gene level expressions but change pathway level mRNA expressions (FDR* q* value < 0.1) are shown in red. In the latter case, there were three mutated genes causing pathway level difference in 24 pathways. The three chosen genes are TP53 (187 are mutated out of 513 samples, 36.4%), PIK3CA (173/513, 33.7%), and RB1 (11/513, 2.1%), reported in breast cancer [14].

It is noteworthy that the pathways of the three genes are addressed as crucial. The three pathways (PI3K, TP53, and RB pathways) are considered as representative pathways for breast cancer [14]. We discovered that mutations on TP53, PIK3CA, and RB1 have significant impact on pathway level mRNA expression, without any prior knowledge, but solely by analyzing the mutation and mRNA expression data. This finding indirectly proves that our approach is sensitive enough to capture the important biological features; thus, it is proper to use our approach to measure pathway level impact of a somatic mutation.

**Figure 4:**
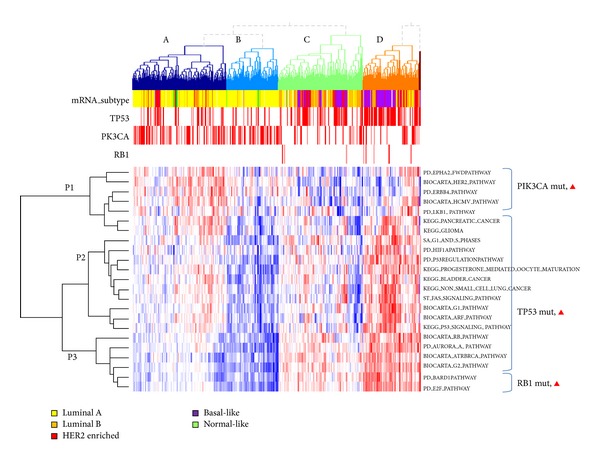
Normalized heatmap illustrating top pathway-influencing mutations (PIK3CA, TP53, and RB1,* q* value < 0.1).

Twenty-four pathways showed differential mRNA expression between groups with and without mutations of TP53, PIK3CA, and RB1 mutations (Figure 4). TP53 and PIK3CA are not mutually exclusive in the observation of TCGA breast cancer data, which is concordant with the previous report [15]. Heatmap visualization of unsupervised clustering of pathway level characteristics shows distinguishing subgroup pattern between “TP53 mutated and PIK3CA non-mutated samples enriched” subgroup (C and D) and “TP53 non-mutated and PIK3CA mutated samples enriched” subgroup (A and B). This characteristic might be explained based on previous findings that TP53 gene product regulates PIK3CA in a transcriptional level [15, 16].

Astanehe et al. [17] demonstrated that direct binding of TP53 reduces the expression of PIK3CA thus decreases the expression of PIK3CA expression. In our analysis, sample cluster of C and D, representing the unmutated samples on PIK3CA, keeps this mRNA deregulation functionality of PIK3CA, showing pathway level downregulation in PIK3CA related pathway cluster P1. Unlike sample clusters C and D, PIK3CA mutation enriched sample cluster of A and B might have TP53 mediated regulation of PIK3CA gene product, showing pathway level upregulation in the pathways P1.

Mutations on RB1 are enriched at sample groups C and D, indicating that it is coupled to TP53 mutation status. Subgroup having mutations on both of RB1 and TP53 has unfavorable outcome when it is compared to others. This observation is concordant with the known biological knowledge that breast cancer subpopulation having retained activity of the major tumor suppressors, RB1 and TP53, has better prognosis than subpopulation of abnormal activity.

In the analysis of impact of single gene mutation on the pathway level mRNA expression change, rare mutations having less than 11 mutated samples were not reported as significant at* q* value < 0.1. To further analyze combinational rare mutation genes in the pathway level mRNA expression, we iteratively combine mutated genes belonging to the pathway so that multiple mutational events on multiple genes can be considered into one mutational event. By combining rare mutational events, we can expect an effect of increasing the sample size of group with mutational event. To reveal rare mutation genes that only work in a combined manner, we did not consider single gene mutations significantly changing pathway level gene expression. This is to avoid false discovery. If a rare mutation with strong influence on the pathway level gene expression is combined with little influence, the latter might be falsely called as significant, due to its influential partner, while it is not truly contributing to the pathway level gene expression change.

**Figure 5:**
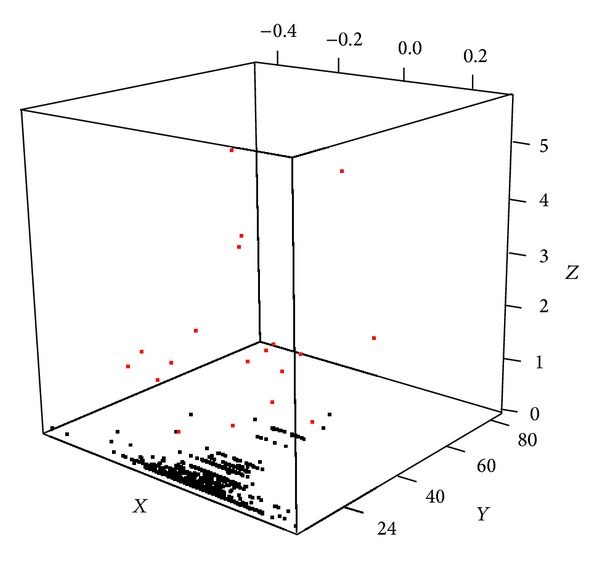
Multiple genes' mutational influence on mRNA expression at pathway level (*X*-axis).* X*: averaged pathway level difference of mutation having group minus nonhaving group.* Y*: number of samples having summarized multigene mutational event.* Z*: −log10*p* score, where* p* is from* t*-test of pathway statistics between mutational event having group versus nonhaving group. Red: mutation event where its influence on pathway level is significant (FDR* q* value < 0.25).

Through our additive combination of rare mutations into one event, we have found 15 mutational events causal on pathway level at the cut of FDR* q* value < 0.1.  Figure 5 depicts the relationship of pathway level mRNA expression difference between groups with and without mutational events (*X*-axis), number of samples having mutational events (*Y*-axis), and the significance of impact of the mutational event (*Z*-axis).

Among 15,819 genes with somatic mutations reported in 513 breast cancer samples, 32 genes were shown to have pathway level impact when mutations on genes are combined as a single mutational event. Gene ontology analysis of these genes using g:Profiler [18, 19] showed significant functional enrichment of these genes into cancer related signalling pathways in biological processes like “fibroblast growth factor receptor signalling pathway” (*P* value = 5.56e−14), “regulation of MAPK cascade” (*P* value = 3.46e−13), “neurotrophin TRK receptor signalling pathway” (*P* value = 2.62e−13), and “ERBB signalling pathway” (*P* value = 1.92E-09). The most significantly enriched gene ontology are “Fc-epsilon receptor signalling pathway” (*P* value = 9.25E−16) for biological process, “Cytosol” (*P* value = 2.21e−07) for cellular compartment and “phosphotransferase activity, alcohol group as acceptor” (*P* value = 1.93e−09) for molecular function. The gene ontology term enrichment analysis provides supportive information that our method does not just coincidentally pick rare mutation genes, but it rather sensitively reveals the additive impact of rare mutations in the context of pathway level mRNA expression change.

**Figure 6:**
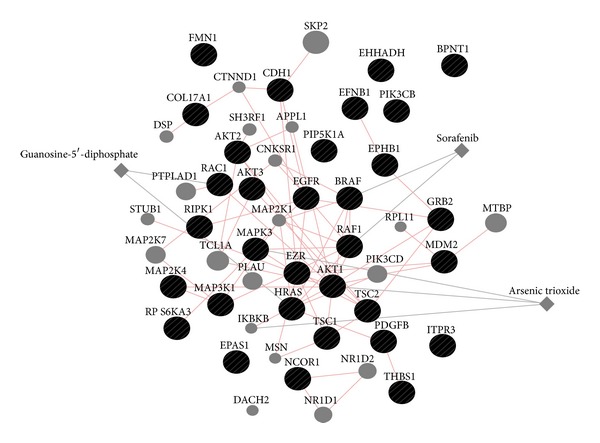
Gene network of 32 genes with pathway level expression change and mutation via GeneMANIA. Two approved drugs (sorafenib and arsenic trioxide) are associated with functional network of 32 genes. Pink edges indicate physical interaction of genes, and grey edges indicate genes that drugs are affecting.

GeneMANIA [20] analysis reveals functional relationships among 32 genes (Figure 6). Two US FDA approved drugs (Sorafenib and Arsenic trioxide) are associated with gene network from 32 genes. Sorafenib has been known to interact with multiple intracellular genes (CRAF, BRAF, and mutant BRAF) and cell surface kinases (KIT, FLT-3, VEGFR-2, VEGFR-3, and PDGFR-*β*). It is a RAF kinase and MAPK pathway inhibitor [21, 22]. A recent study reported sorafenib benefited patients with RAS and BRAF mutations [23]. According to clinicaltrials.gov, twenty-five clinical trials are on-going for sorafenib treatment on breast cancer in the US. The collected information is supportive of the 32 genes prioritized by our method is functionally important in cancer; thus, it should be clinically considered for targeted therapeutics.

The 32 genes are compared to the lists of significantly mutated genes from the previous breast cancer sequencing studies. The TCGA study yielded 23 significantly mutated genes from 509 patients [14]. Ellis and Perou used the same data to define 28 significantly mutated genes important in therapeutic consideration in breast cancer [24]. Banerji et al. studied 103 breast cancer patients in Mexico and Vietnam and reported 6 significantly mutated genes [25]. Among the 32 genes we discovered, there are 5 overlaps to the previously reported genes (Supplementary Figure 2, Supplementary Table 1), and 27 genes do not belong to any gene list from the previous breast cancer studies. In the previous studies, significantly mutated genes were defined as mutations observed at higher frequency than expected at random. In our analysis, we find a list of genes particularly with mutational events significantly changing the pathway level mRNA expression. We think that, due to this methodological difference, the findings of our study should be interpreted with a different biological point of view. In other words, the discovered genes have a unique interpretation in the context of pathway level mRNA change that has not been addressed in other studies.

Based on these results, we suggest the 32 genes, along with 3 genes that showed single gene's mutational influence on pathways as potential tumor driver mutations, to have more functional importance than the other 15,750 somatic mutations.  Table 2 provides mutational events of 32 genes that have shown pathway level impact in a combinatorial manner.

We further investigated pathway level impact of somatic mutations against gene networks with actionable drugs. Among 19 receptor tyrosine-kinase related pathways, three pathways have shown pathway level difference with two mutational events (Table 3).

LYN is a member of src kinase superfamily and is known to be involved in the regulation of cell activation. Ellis and Perou [24] addressed that SH2 domain missense mutation D197Y at breast cancer is functional. Overexpression of D197Y is more potent than wild type LYN at inducing signalling cascade, rendering the treatment of ER downregulator fulvestrant or PI3K inhibitor BKM120 less effective. This indicates that LYN may play a role for ER+ breast cancer acquiring hormone-independent growth. Two LYN mutations in our discovery for RTK pathway related mutational event were also located SH2 domain (E159 K, K188N). The two mutations may be considered to have similar contribution to D197Y.

NCK1 is downstream of signal cascade of LYN. Its major function is activating actin cytoskeleton reorganization. However, there is no documentation on how LYN and NCK1 regulate the transcription level of PDGFR pathways. Our observation indicates that the group with either mutation of the two genes has lower level of gene expression in the PDGFR pathway than the group without mutations.

Among mutational event of 16 samples on any of the three genes (PIK3R1, PIK3CD, and GRB2), PIK3R1 is the most frequent (number of event samples: 14). Most of the 14 mutations are clustered in the PIK3CA interaction domain. BKM120 and GDC-0941 are the suggested drugs for patients having mutation at PIK3R1 sites [14]. PIK3R1, PIK3CD, and GRB2 all interact together in a protein level. This suggests that mutation on any of these genes can cause similar functional impact on downstream pathways.

In summary of receptor tyrosine kinase pathways, we additionally discovered two mutational events that have significant pathway level mRNA change. Literature survey [21–23, 26] on discovered mutations also revealed that the mutations are potential drug targets. This is supportive evidence that our method can sensitively detect functional rare mutations; in other words, measuring pathway level impact of summarized rare mutational events is useful to prioritize the functional ones.

## 4. Conclusion

In this paper, we propose a practical approach that assesses mutational impact on pathway level mRNA expression. We suggest combinatorial summary of mutational events. We have demonstrated that the proposed approach sensitively discovers important mutations that have been known to have pathway level functional impact. Important mutations that cause deregulation of representative breast cancer pathways reported by previous study have been captured by our approach.

Combinational mutation summary found 32 genes that showed pathway level difference between the two groups, with and without mutational events. Gene ontology enrichment test of the 32 genes shows significant enrichment in the cancer-related biological processes such as “MAPK cascade,” “ERBB2 signalling,” “Fibroblast growth receptor signaling,” suggesting that the combinational mutational summary captures are actually involved in cancer mechanism. Based on the pathway level impact analysis result, we suggested that the functional importance of somatic mutations on the 32 genes is bigger than that of the others.

We also investigated impact of rare mutations on drug target pathways; we found two mutational events that consisted of two and three genes. Two of total five mutations were mentioned as potential drug target in the literature, indirectly supporting that our approach is useful to prioritize druggable mutations.

Due to the innovation of next generation sequencing technology, more cancer patients' genomic and transcriptomic data are expected to be available. We hope that our proposed approach can be used to discover mutations having functional impact. The approach can further be used to prioritize mutations for the consideration of custom tailored therapy.

## Supplementary Material

Supplement Figure 1. The schematic diagram of the Pathway-driven discovery of rare mutational impact on cancer. A diagram describes the ‘while' loop part in the pseudo-code.
Supplement Figure 2. Comparing mutated genes showing pathway level mRNA difference (Discovered) to genes reported as significantly mutated in breast cancer. Supplement table 1 provide detailed information.
Supplement Table 1. A list of mutated genes showing pathway level mRNA difference (Discovered) and genes previously reported as significantly mutated in breast cancer.
Supplement Table 2. A list of abbreviations (alphabetical order).

## Figures and Tables

**Algorithm 1:**
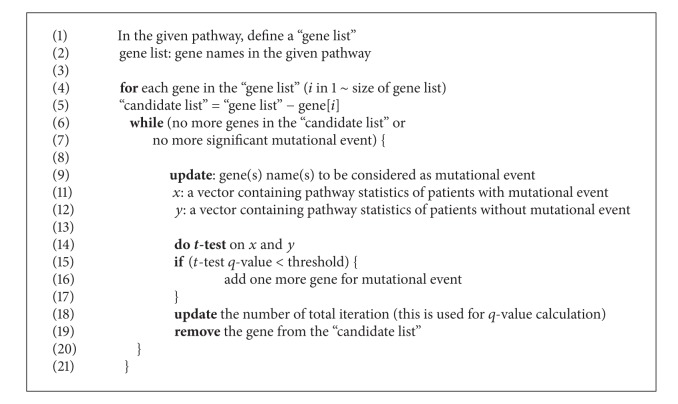


**Table 1:** Curated drug target centric pathways.

Target	Drugs	First neighbors
EGFR	cetuximab, AEE 788, panitumumab, BMS-599626, ARRY-334543, XL647, canertinib, gefitinib, HKI-272, PD 153035, lapatinib, vandetanib, erlotinib	125
PDGRFB	dasatinib, sunitinib, pazopanib, axitinib, KRN-951, tandutinib, imatinib, sorafenib, becaplermin	61
ERBB2	trastuzumab, BMS-599626, ARRY-334543, XL647, CP-724,714, HKI-272, lapatinib, erlotinib	59
MET	crizotinib	55
ERBB4	BMS-599626	44
KIT	dasatinib, sunitinib, pazopanib, KRN-951, OSI-930, telatinib, tandutinib, imatinib, sorafenib	38
FLT4	sunitinib, pazopanib, CEP 7055, KRN-951, telatinib, sorafenib, vandetanib	36
PDGFRA	sunitinib, pazopanib, axitinib, telatinib, imatinib, becaplermin	35
TEK	Vandetanib	35
RET	sunitinib, vandetanib	30
FGFR1	Pazopanib	29
EPHA2	Dasatinib	22
FGFR3	Pazopanib	18
FLT3	CHIR-258, tandutinib, sorafenib, lestaurtinib, CGP 41251	14
FGFR2	Palifermin	13

**Table 2:** List of multi-gene mutational events with pathway level expression change.

Pathway	Mutational event (# of distinct genes: 32)	# event sample	*t*-stat	*q*-value
KEGG_GLIOMA	PIK3CB, HRAS	5	12.587	0.000
KEGG_MELANOMA	PIK3CB, BRAF	7	10.302	0.000
PID_CDC42	CDH1, MAP3K1	70	−5.244	0.000
PID_TRAIL	RIPK1, MAPK3	6	9.624	0.000
BIOCARTA_PPARA	NCOR1, EHHADH	21	−4.562	0.007
PID_A6B1_A6B4_INTEGRIN	COL17A1, GRB2	6	−6.239	0.009
BIOCARTA_MTOR	TSC1, TSC2	7	−5.081	0.010
SIG_PIP3_SIGNALLING_IN_B_LYMPHOCYTES	ITPR3, RPS6KA3	7	6.174	0.014
PID_CERAMIDE	MAP2K4, AKT1, RIPK1	35	4.803	0.017
SA_PTEN	AKT3, BPNT1	5	−6.573	0.018
ST_FAS_SIGNALLING	MAP3K1, EZR	42	−3.727	0.029
PID_EPHBFWDPATHWAY	EPHB1, EFNB1	9	−4.848	0.032
KEGG_RENAL_CELL_CARCINOMA	EPAS1, GRB2, PDGFB	7	7.628	0.057
PID_ECADHERIN_KERATINOCYTE	CDH1, FMN1, PIP5K1A, EGFR, AKT2, CDH1, RAC1, CDH1, RAC1	49	−5.564	0.074
KEGG_BLADDER_CANCER	CDH1, THBS1, MDM2, RAF1	41	−4.966	0.083

**Table 3:** List of multi-gene mutational events with pathway level expression change on drug target centric pathways.

Pathway	Mutational event	# event samples	*t*-stat	*q*-value
PDGFRB_neighbors	LYN, NCK1	5	−8.3139	0.005
FGFR2_neighbors	PIK3R1, PIK3CD, GRB2	18	3.506	0.141
