# Supplementary material for: Pathway-Driven Discovery of Rare Mutational Impact on Cancer
Source: Biomed Res Int. 2014 May 4;2014:171892. doi: 10.1155/2014/171892 (PMC4026869; doi:10.1155/2014/171892)

Supplement Figure 1. The schematic diagram of the Pathway-driven discovery of rare mutational impact on cancer. A diagram describes the ′while′ loop part in the pseudo-code.

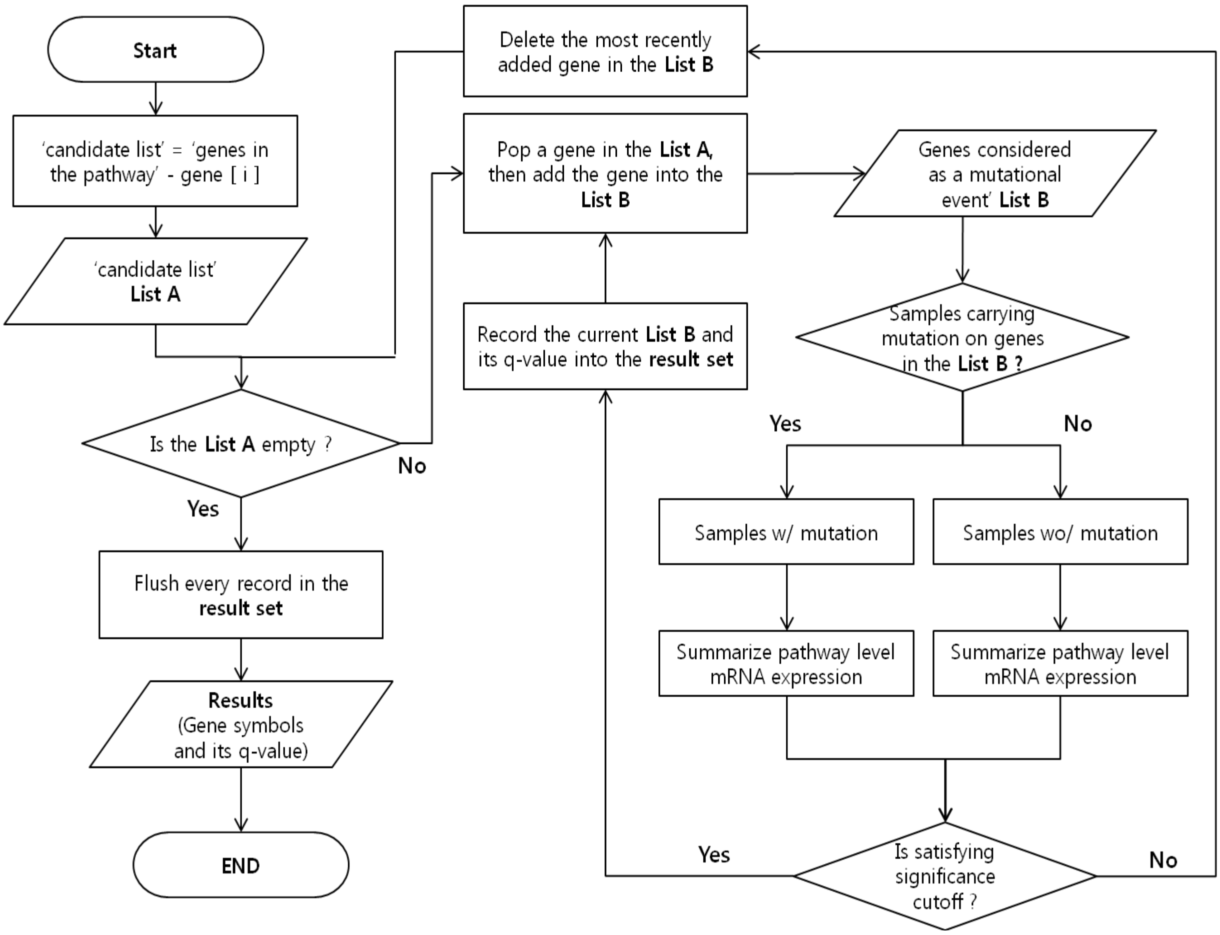

Supplement: Supplementary file 1 — Supplement Figure 1. The schematic diagram of the Pathway-driven discovery of rare mutational impact on cancer. A diagram describes the ‘while' loop part in the pseudo-code. Supplement Figure 2. Comparing mutated genes showing pathway level mRNA difference (Discovered) to genes reported as significantly mutated in breast cancer. Supplement table 1 provide detailed information. Supplement Table 1. A list of mutated genes showing pathway level mRNA difference (Discovered) and genes previously reported as significantly mutated in breast cancer. Supplement Table 2. A list of abbreviations (alphabetical order). [file 171892.f1.pdf]
