# Supplementary material for: Pathway-Driven Discovery of Rare Mutational Impact on Cancer
Source: Biomed Res Int. 2014 May 4;2014:171892. doi: 10.1155/2014/171892 (PMC4026869; doi:10.1155/2014/171892)
Supplement: Supplementary file 2 [file 171892.f2.pdf]

Supplement Figure 2. Comparing mutated genes showing pathway level mRNA difference (Discovered) to genes reported as significantly mutated in breast cancer. Supplement table 1 provide detailed information.

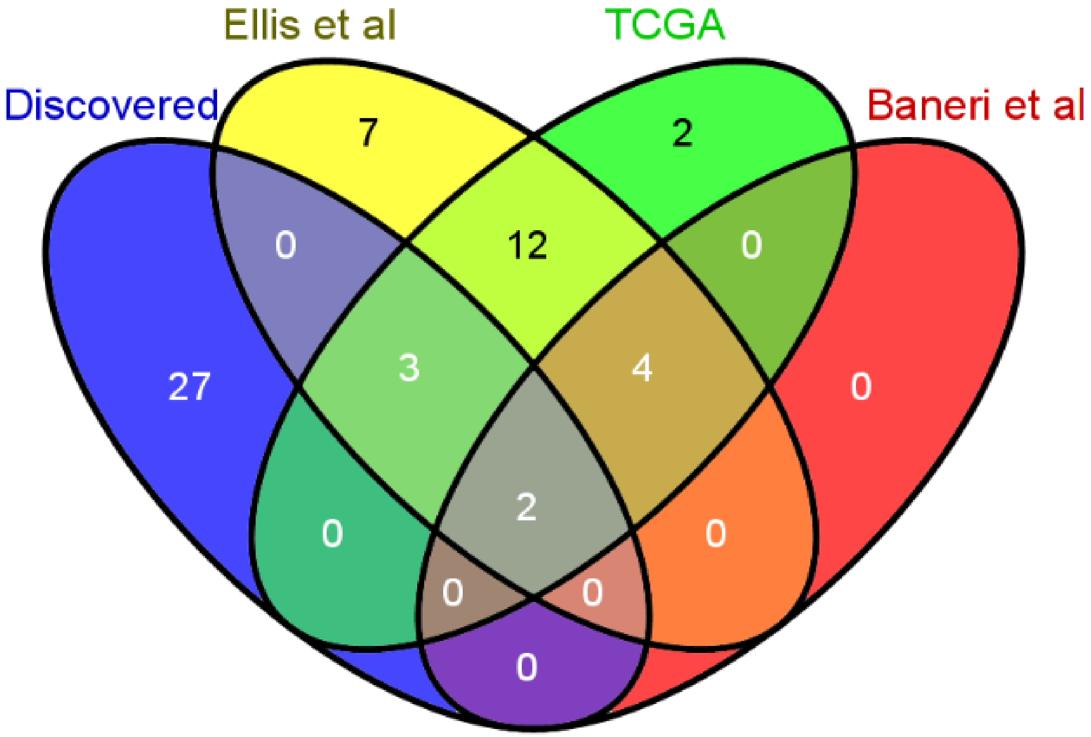

Ellis, M. J. & Perou, C. M. (2013) 'The genomic landscape of breast cancer as a therapeutic roadmap', Cancer Discov, 3, 27-34.

TCGA (2012) ‘Comprehensive molecular portraits of human breast tumours’, Nature, 490, pp.61-70.

Banerji, S., Cibulskis, K., Rangel-Escareno, C., Brown, K. K., Carter, S. L., Frederick, A. M., et al. (2012) 'Sequence analysis of mutations and translocations across breast cancer subtypes.\', Nature, 486, 405-409.
