# Supplementary material for: Pathway-Driven Discovery of Rare Mutational Impact on Cancer
Source: Biomed Res Int. 2014 May 4;2014:171892. doi: 10.1155/2014/171892 (PMC4026869; doi:10.1155/2014/171892)
Supplement: Supplementary file 3 [file 171892.f3.pdf]

Supplement Table 1. A list of mutated genes showing pathway level mRNA difference (Discovered) and genes previously reported as significantly mutated in breast cancer

Discovered	Ellis et al.,	TCGA	Baneri et al.,
AKT1	AKT1	PIK3CA	TP53
AKT2	CBFB	TP53	PIK3CA
AKT3	CDH1	MAP3K1	AKT1
BPNT1	CDKN1B	MAP2K4	CBFB
BRAF	CTCF	GATA3	GATA3
CDH1	FAM47C	MLL3	MAP3K1
COL17A1	FOXA1	CDH1	
EFNB1	GATA3	PTEN	
EGFR	GPS2	PIK3R1	
EHHADH	MAP2K4	AKT1	
EPAS1	MAP3K1	RUNX1	
EPHB1	MLL3	CBFB	
EZR	NCOR1	TBX3	
FMN1	NF1	NCOR1	
GRB2	OR2L2	CTCF	
HRAS	OR6A2	FOXA1	
ITPR3	PIK3CA	SF3B1	
MAP2K4	PIK3R1	CDKN1B	
MAP3K1	PTEN	RB1	
MAPK3	PTPN22	AFF2	
MDM2	RB1	NF1	
NCOR1	RUNX1	PTPN22	
PDGFB	SF3B1	PTPRD	
PIK3CB	TBL1XR1		
PIP5K1A	TBX3		
RAC1	TLR4		
RAF1	TP53		
RIPK1	ZFP36L1		
RPS6KA3			
THBS1			
TSC1			
TSC2			

Ellis, M. J. & Perou, C. M. (2013) 'The genomic landscape of breast cancer as a therapeutic roadmap', Cancer Discov, 3, 27-34.
TCGA (2012) ‘Comprehensive molecular portraits of human breast tumours’, Nature, 490, pp.61-70.
Banerji, S., Cibulskis, K., Rangel-Escareno, C., Brown, K. K., Carter, S. L., Frederick, A. M., et al. (2012) 'Sequence analysis of mutations and translocations across breast cancer subtypes.\', Nature, 486, 405-409.
