# Supplementary material for: Pathway-Driven Discovery of Rare Mutational Impact on Cancer
Source: Biomed Res Int. 2014 May 4;2014:171892. doi: 10.1155/2014/171892 (PMC4026869; doi:10.1155/2014/171892)
Supplement: Supplementary file 4 [file 171892.f4.pdf]

Supplement Table 2. A list of abbreviations (alphabetical order)

Abbreviation	Description
CNV	Copy Number Variation
FCS	Functional Class Scoring
FDA	Food and Drug Administration
FDR	False Discovery Rate
iPAS	individualized Pathway Aberrance Score
ORA	Over-Representing Analysis
PARADIGM	PAthway Recognition Algorithm using Data Integration on Genomic Models
PCA	Principal Component Analysis
PDS	Pathway Deregulation Score
RTK	Receptor Tyrosine Kinase
TCGA	The Cancer Genome Atlas

"The authors declare that there is no conflict of interests regarding the publication of this article."
